# Supplementary material for: Risk Stratification for Immediate Postoperative Hearing Loss by Preoperative BAER (Brainstem Auditory Evoked Response) and Audiometry in NF2-Associated Vestibular Schwannomas
Source: Cancers (Basel). 2021 Mar 18;13(6):1384. doi: 10.3390/cancers13061384 (PMC8003145; doi:10.3390/cancers13061384)
Supplement: Supplementary file 1 [file cancers-13-01384-s001.pdf]

cancers

MDPI

Supplementary Materials: Risk Stratification for Immediate Postoperative Hearing Loss by Preoperative BERA and Audiometry in NF2 associated Vestibular Schwannoma

Isabel Gugel, Florian Grimm, Philip Hartjen, Maria Breun, Julian Zipfel, Marina Liebsch, Hubert Löwenheim, Ulrike Ernemann, Lan Kluwe, Victor-Felix Mautner, Marcos Tatagiba and Martin Ulrich Schuhmann

Figure S1–S7. Exemplary long-term hearing function and tumor volume courses of discrepant cases between AAO–HNS and BAEP classification.

Note for all figures. MRI—magnetic resonance imaging; vol—volume; PTA—pure-tone average in decibel (dB); SDS—speech discrimination score in %; BAEP—brainstem auditory evoked potentials Classification System according to Samii and Matthies et al. [1]; AAO–HNS Classification—American Association of Otolaryngology–Head and Neck Surgery [2].

Tumor size was measured in volume (cm^3). Blue and orange dots represent the measurement for the left and right vestibular schwannoma (VS), respectively. Vertical blue and orange lines indicate the surgery times for the left and the right VS, respectively.

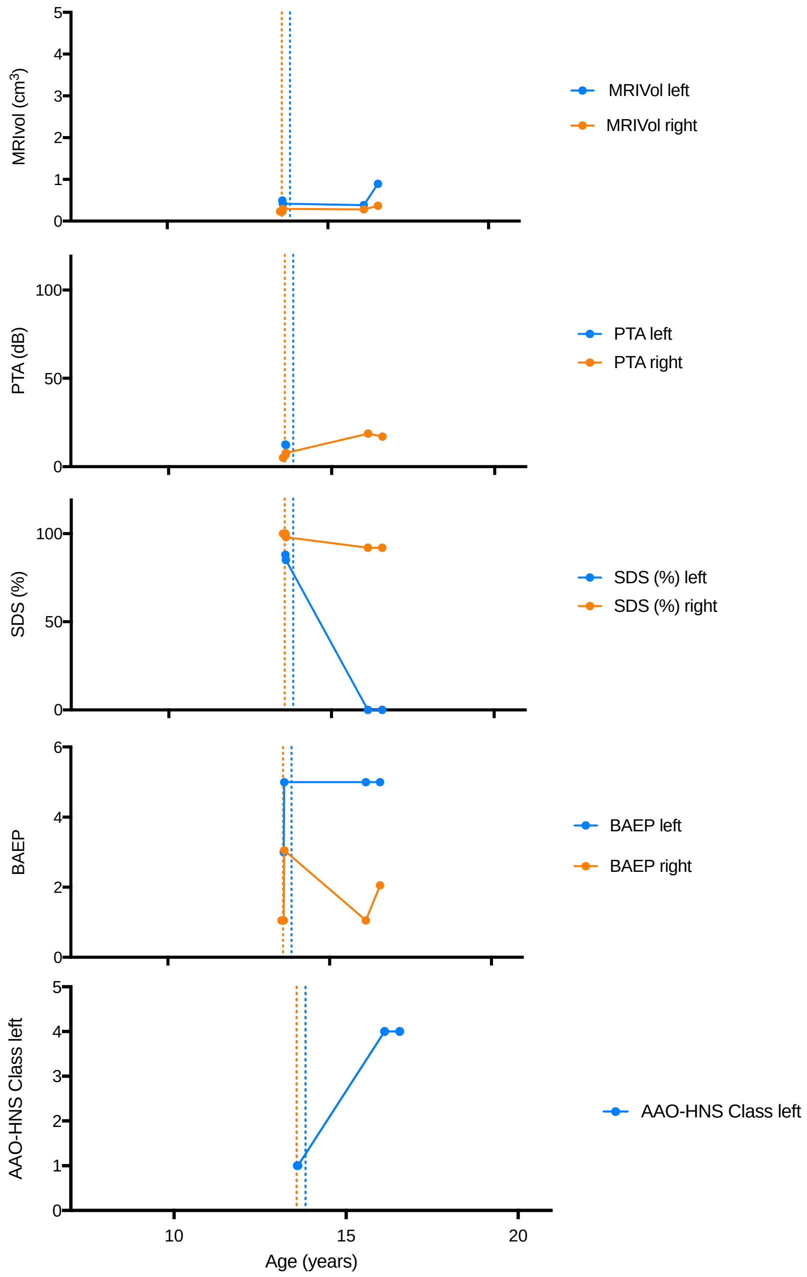

Figure S1. (case 1.1 left tumor). The mean follow-up was 3 months before and 32 months after surgery. Values at last follow-up: volume: 0.89 cm^3; PTA: 130 dB; SDS: 0%.

Both VS were small (T1) at first presentation in our department. The first surgery on the right VS was performed quickly three months after first presentation due to a beginning decrease in BAEP left more than right. Three months after, indication for surgery was given on the left due to a rapid drop in BAEPs from class 3 to 5 within two weeks but still normal PTA and SDS values (AAO–HNS class 1). For facial preservation and due to intraoperatively fragile MEPs of the facial nerve after starting to enucleate the tumor near the facial nerve, only a small resection amount was achieved. Directly after surgery the patient was completely deaf.

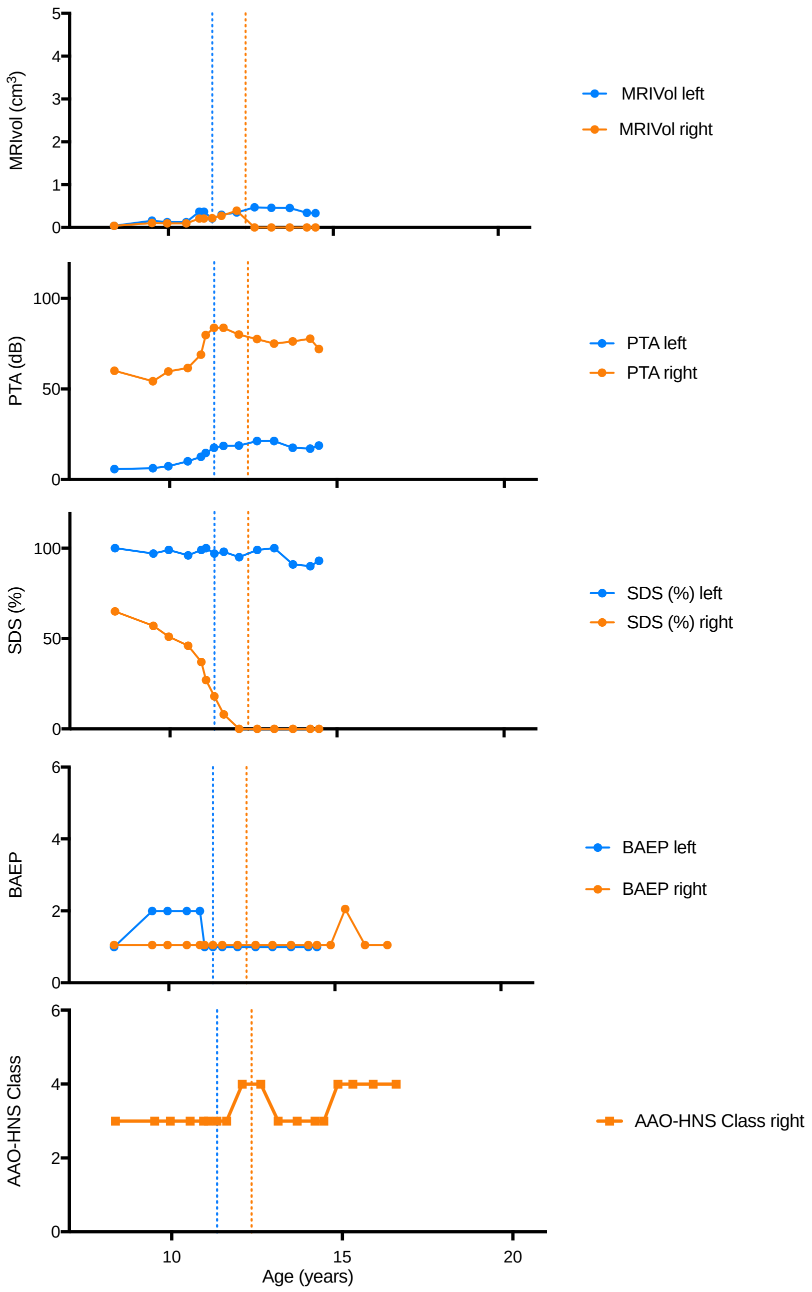

Figure S2. (case 2.2 right tumor). The mean follow-up was 48 months before and 53 months after surgery. Values at last follow-up: volume: 0 cm^3; PTA: 76.75 dB; SDS: 30%.

This initially seven-year-old female patient presented with a severe hearing deterioration on the right ear (PTA 85 dB, SDS 30%) which had led to further clarification and diagnosis of the NF2 disease. The left hearing was normal and bilateral VS were small. In contrast to audiometric results, brainstem auditory evoked potentials (BAEPs) were bilaterally normal. Initially, hearing-preserving surgery was performed on the normal hearing left ear. After a follow-up period of 48 months, a beginning decrease in BAEP parallel to tumor volume increase up to 0.39 cm^3 on the right side, indication for surgery was given. Due to the poor hearing, the surgical goal was total tumor removal aiming at preservation of the cochlear nerve, to be able to receive a cochlear implant in case hearing of the other side would get lost in the following course. Total tumor removal was achieved with endoscope assisted intracanalicular surgery at the fundus with preservation of cochlear and facial nerve. Intraoperatively, BAEPs were lost; however, started to recover at the time of

skin closure. Interestingly, right after surgery, the patient's hearing capacity of this ear notably improved. Further improvement was seen in BAEPs and audiometry three months after surgery. Magnetic resonance imaging (MRI) confirmed complete resection of the right-sided tumor. Recently, the patient received a cochlea implant (CI) on the right ear and is now planned for radiation due to a small tumor relapse on that ear in order to maintain the good CI assisted hearing by preventing further tumor growth.

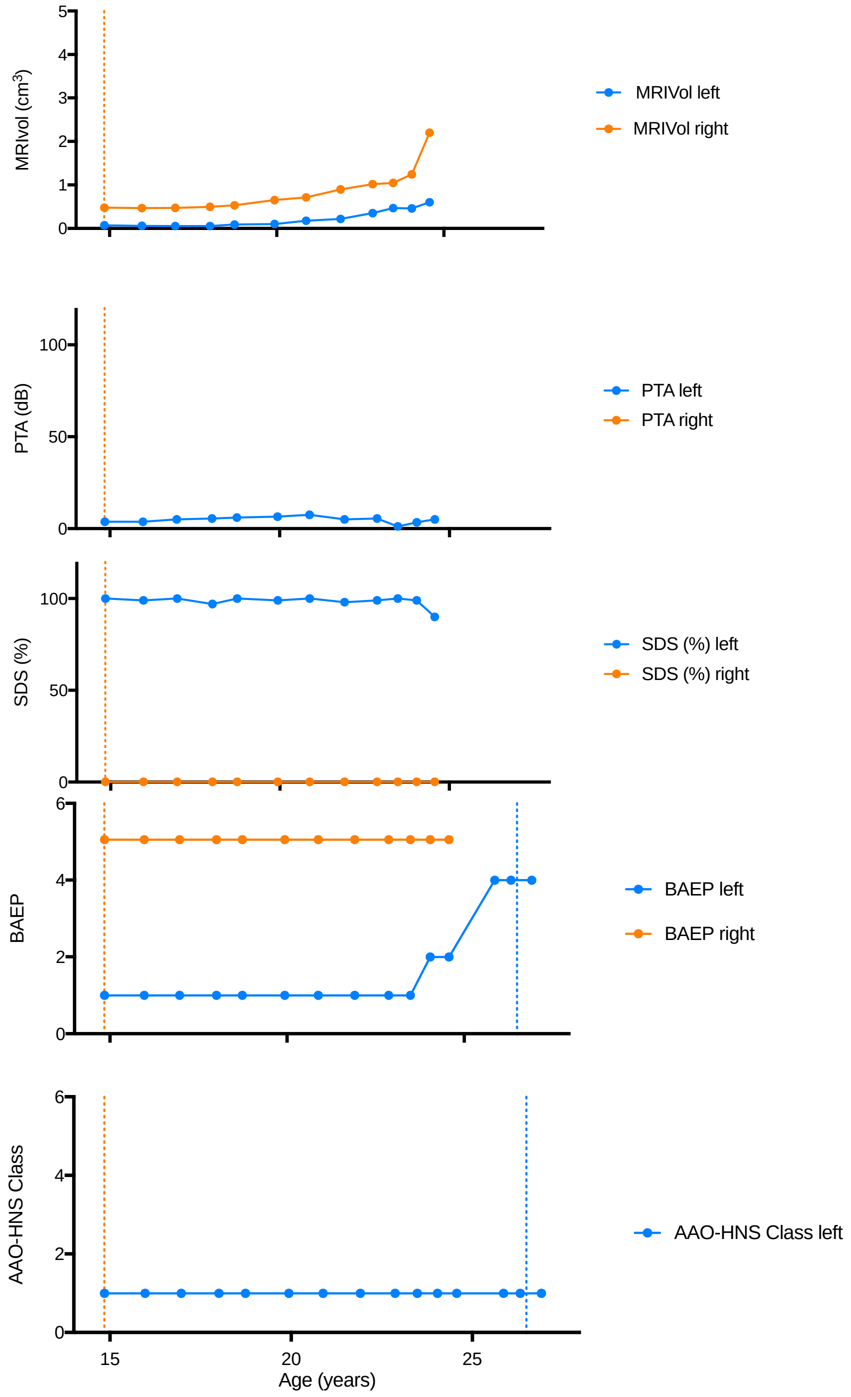

Figure S3. (case 3.1 left tumor). The mean follow-up was 139 months before and 5 months after surgery. Values at last follow-up: volume: 1.54 cm^3; PTA: 7.5 dB; SDS: 95 %.

Initially, this female patient was near totally resected in an external institution on the right side and was consecutively postoperatively deaf. After 132 months of follow-up with stable hearing and small tumor volumes on the left side, BAEP deteriorated from class 1 to 4 and tumor volume increased while AAO-HNS class remained stable and normal (class 1). After partial resection (approximately 50%), the tumor volume increased in the first postoperative control after three months whereas hearing remained stable. The residual VS on the right side was accompanied by a huge trigeminal nerve schwannoma and both tumors were growing with a relevant brainstem compression so that another surgery in form of a total resection of VS and subtotal resection of the trigeminal nerve schwannoma was performed and a test stimulation for an auditory brainstem implant was tried but no stimulation could be achieved after 13 years of deafness. To stabilize hearing and tumor growth on the remaining hearing left ear, bevacizumab is now initiated.

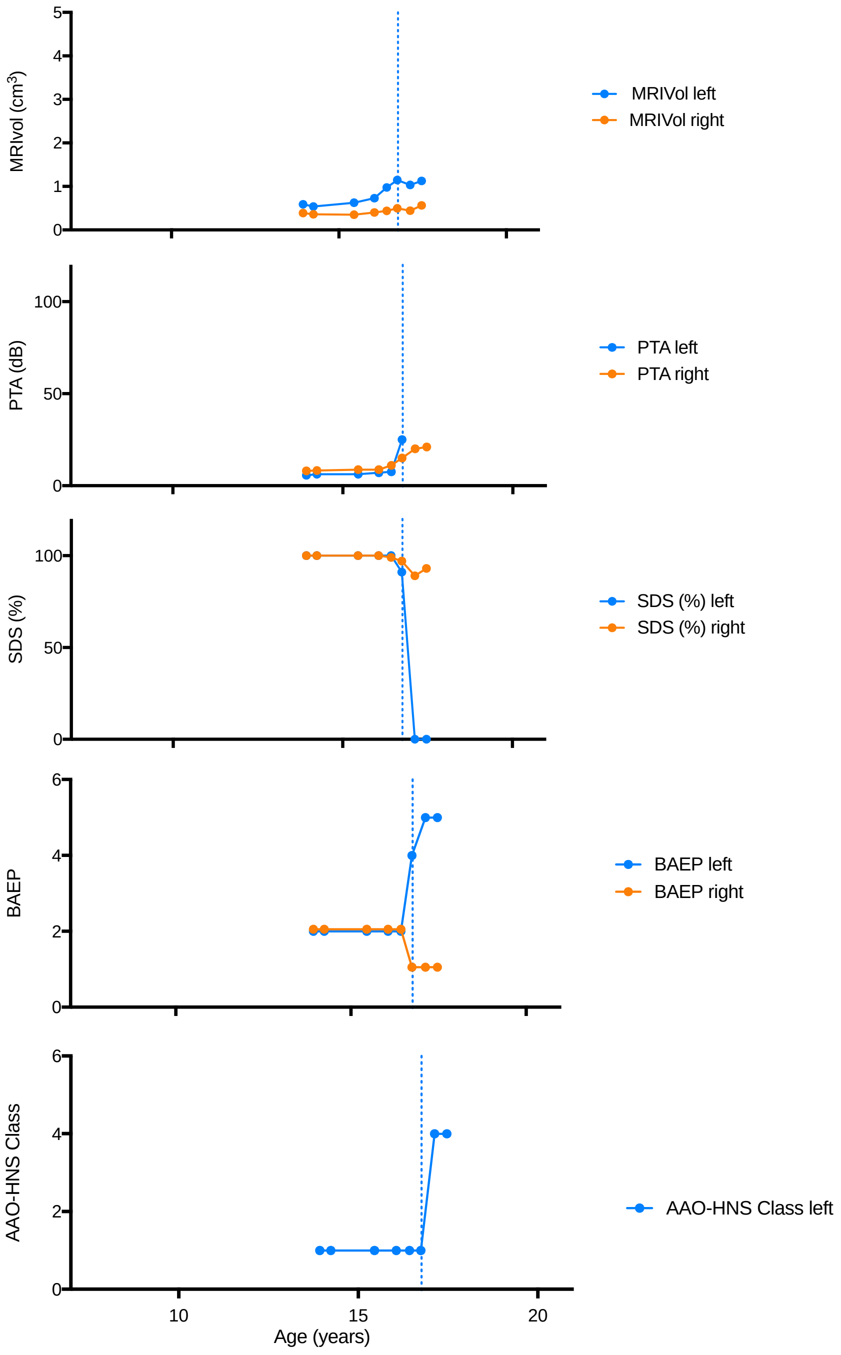

Figure S4. (case 4.1 left tumor). The mean follow-up was 34 months before and 8 months after surgery. Values at last follow-up: volume: 1.13 cm³; PTA: 130 dB; SDS: 0 %.

Young female patient with NF2 diagnosis establishment at the age of 17 and first symptoms (cutaneous plexiform schwannoma, idiopathic anisocoria) at the age of 13. She presented herself at our department at the age of 14 and a hearing decline on the left side began at the age of 15. Therefore, surgery was indicated but initially refused from the patient. A further hearing deterioration led to a therapy, 2 years later, and thus was performed after 34 months of observation and when BAEP decrease from class 2 to 4 while AAO-HNS class remained stable (class 1). After drilling of the IAC the BAEPs started to undulate and decrease at the beginning of opening the tumor capsule and remained unstable. Therefore, only a very small sample was carefully taken. The BAEPs recovered to the preop value at the end of surgery. Directly after surgery the patient was totally deaf. The remaining hearing right ear remained stable until the last follow-up at our department in 2017 with a change of the NF2 Center.

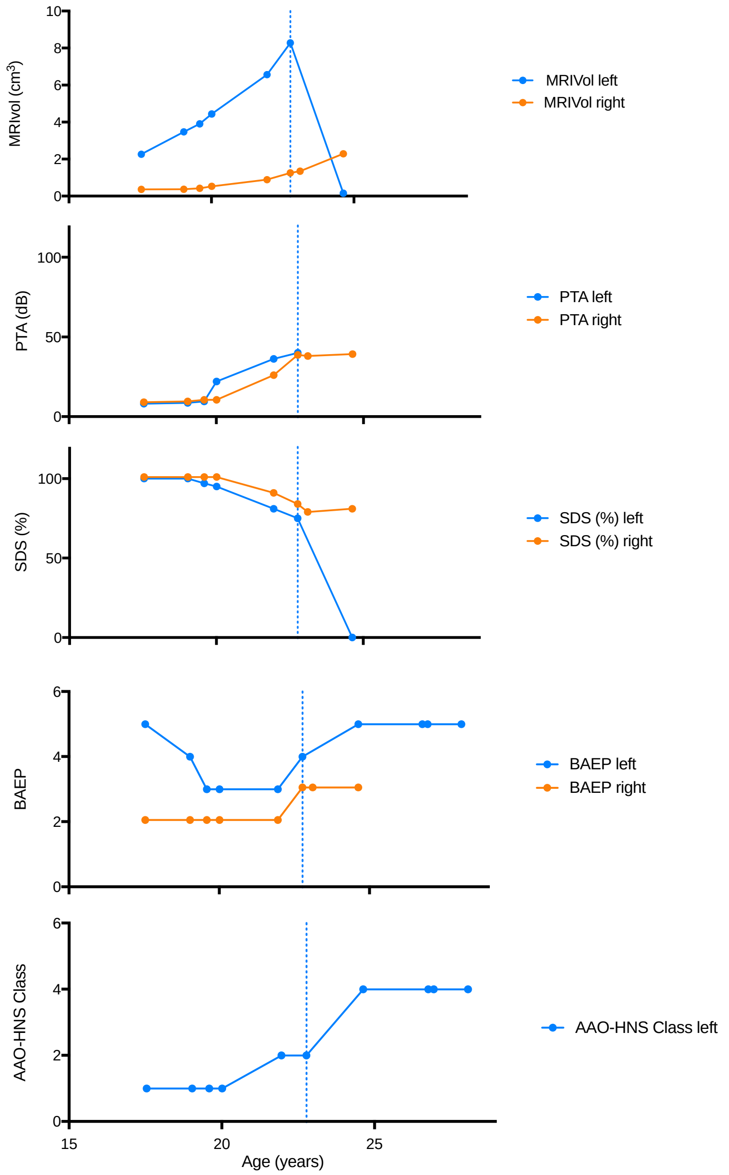

Figure S5. (case 5.1 left tumor). The mean follow-up was 63 months before and 63 months after surgery. Values at last follow-up: volume: 3.85 cm³; PTA: 130 dB; SDS: 0 %.

Male patient with a positive family history for NF2 (father and brother) and first presentation with a subarachnoid hemorrhage due to a ruptured pericallosal artery aneurysm at the age of 17 years. The initial MRI scan showed a T2 VS right and a T3a VS on the left side. In the following course the larger left sided tumor was clearly with a class T4a before surgery but stable and normal hearing on both side sand surgery to decompress the brainstem was indicated. Meanwhile within three months until date of surgery, the hearing on the left side severely deteriorated compared to the better hearing right side at that time. BAEPs 3 months before surgery were worse (BAEP 4) but still available. Intraoperatively, they were no more reproducible under anesthesiologic conditions. Surgery in form of a decompression of the IAC and subtotal resection under preservation of the facial nerve was performed, but the cochlear nerve, which was anatomically preserved but extremely thinned out due to the tumor. The patient was totally deaf after the surgery.

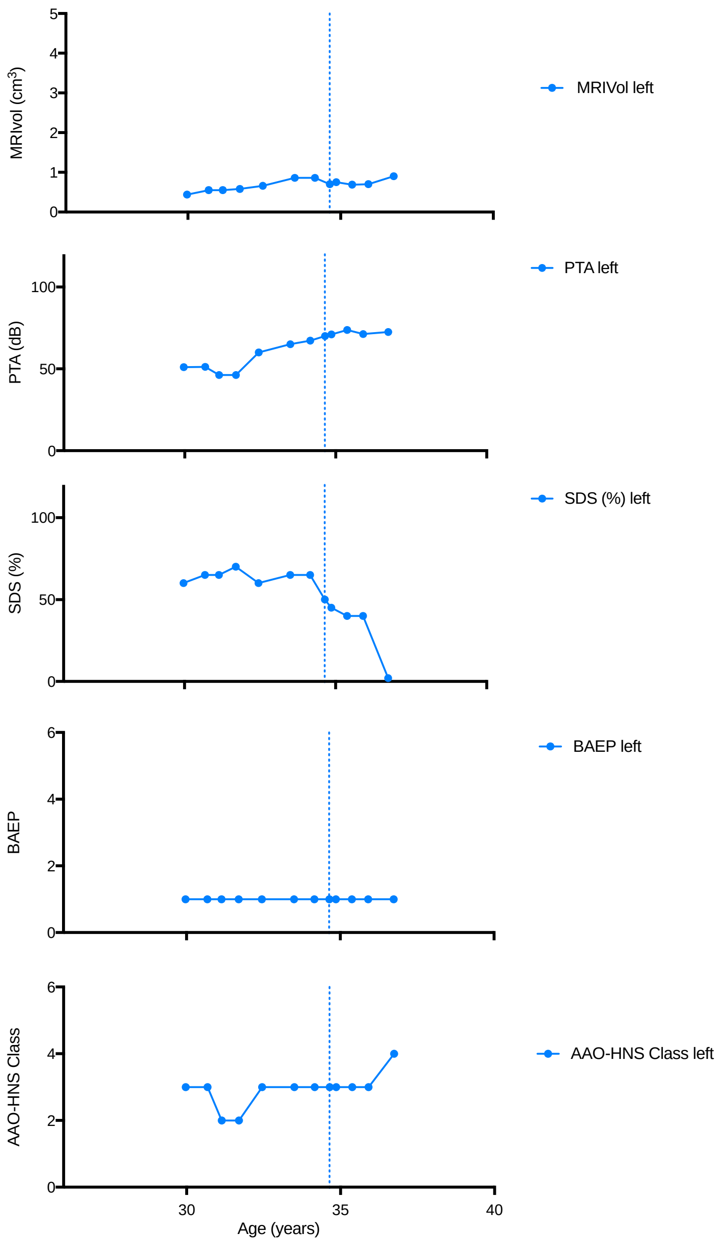

Figure S6. (case 6.1 left tumor). The mean follow-up was 57 months before and 10 months after surgery. Values at last follow-up: volume: 0.9 cm^3; PTA: 72.5 dB; SDS: 2 %.

Another male patient with a positive family history for NF2 (daughter) who presented and was diagnosed late with functional deafness before and after surgery. Due to incompliance and refuse of a surgical procedure on the better but declining hearing on the left side, surgery was performed four years later to the initial presentation at our department and with a worse hearing state combined with tumor progression. BAEPs before surgery were nearly no more reproducible but available for intraoperative monitoring. After decompression of the IAC and beginning of tumor debulking, BAEPS were unstable but fully recover after several stops. Nevertheless, after approximately 40% of resection amounts, surgery was stopped to prevent a possible and remaining drop of BAEPs. Functional hearing directly after surgery was identical to the preoperative impression. BAEPs were slightly better in the BAEP control before discharge.

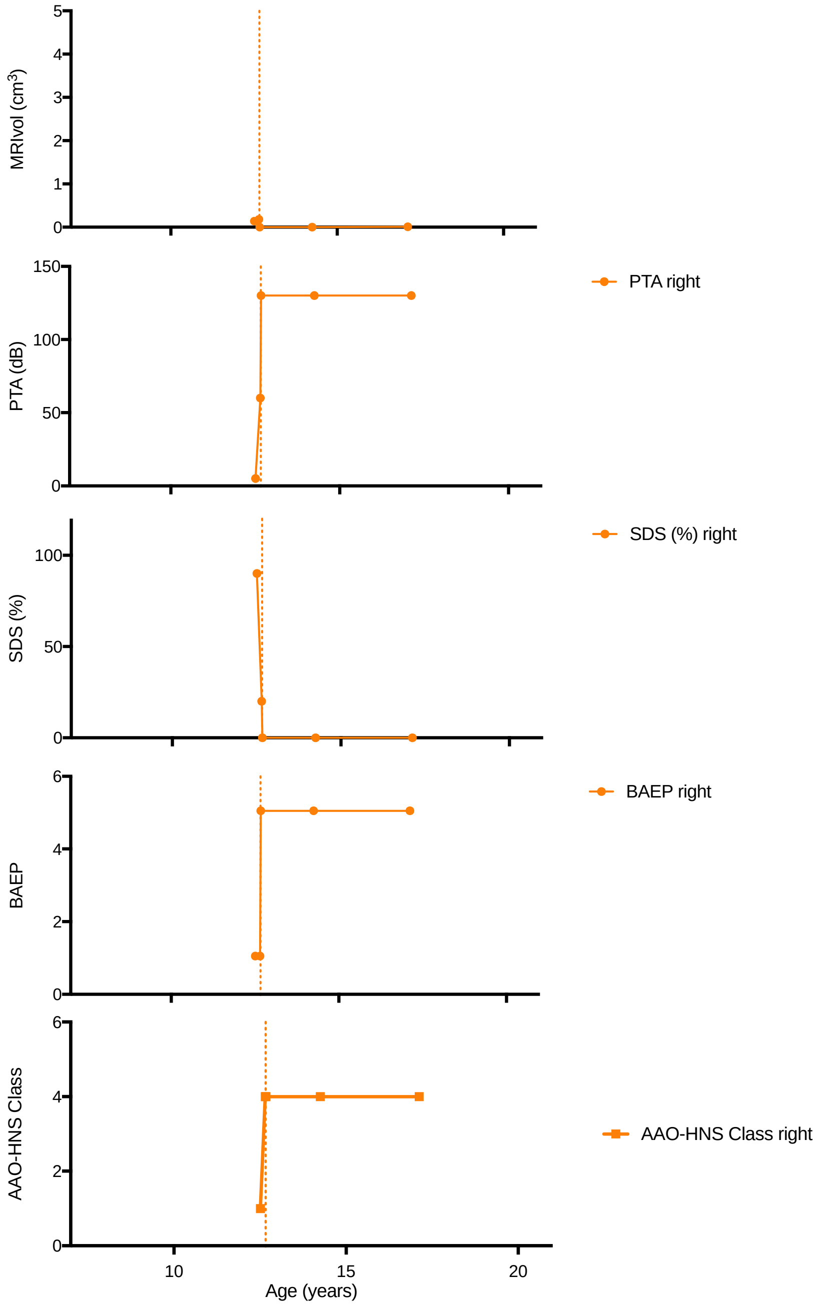

Figure S7. (case 7.2 right tumor). The mean follow-up was 2 months before and 53 months after surgery. Values at last follow-up: volume: 0.01 cm^3; PTA: 130 dB; SDS: 0 %.

This male NF2 patient presented himself with small VS (T1) and a medium but progressive hearing loss on the right side and normal hearing on the left side. Surgery was performed on the right side with total tumor resection. Intraoperatively and at the beginning of the surgery, BAEPs were severely unstable. Anatomically, the cochlear nerve was preserved but hearing directly after surgery was completely gone. The patient is no longer in regular follow-ups at our department.

Table S1. Parameters for the 100 operated vestibular schwannomas from 72 Neurofibromatosis Type 2 patients.

Tumor/ Patient ID	Sex	Tumor Side	Mutation Type	Family History of NF2	Age at NF2 Diagnosis in Years	Age at Time of Surgery in Years	Preoperative					Postoperative					Resection Amount in % (Calculated with Volumes)	Resection Amount Category*
							PTA in dB	SDS in %	BAEP Class	Growth Rate in cm³/Year	Volume in cm³	PTA in dB	SDS in %	BAEP Class	Growth Rate in cm³/Year	Volume in cm³		
1.1	F	L	NS	Neg	3	13	12.25	85	5	−0.18	0.42	130	0	5	1.16	0.38	8	2
1.2	-	R	-	-		13	5	100	1	NA	0.23	6.5	100	1	0.06	0.16	30	3c
2.1	F	L	NS	Neg	7	11	14.7	100	1	0.12	0.37	17.5	97	1	0.03	0.21	44	3c
2.2	-	R	-	-		12	80	30	1	0.09	0.39	77.5	60	1	0.02	0.00	100	6
3.1	F	L	NA	Neg	15	15	10	100	4	0.09	1.16	7.5	95	4	NA	1.54	−32	7
4.1	F	L	NA	Neg	13	16	25	91	4	0.20	1.15	130	0	5	0.26	1.04	10	3b
5.1	M	L	FS	Pos	17	22	40	75	4	1.18	8.28	130	0	5	1.14	0.16	98	5
6.1	M	L	MOS	Pos	30	34	67.25	65	1	0.10	0.86	70	50	1	0.07	0.70	19	3b
7.2	M	R	NA	Neg	12	12	60	20	1	0.30	0.18	130	0	5	0.00	0.00	100	6
8.1	M	L	NO	Neg	8	12	10	100	1	0.00	0.23	35	82	2	0.47	0.13	43	3c
8.2	-	R	-	-		8	28.75	100	1	NA	2.20	28	97	2	0.53	1.58	28	3b
9.1	F	L	FS	Neg	10	12	5	100	2	0.14	0.23	5	100	2	0.15	0.21	9	2
9.2	-	R	-	-		16	5	100	1	0.07	0.39	6.25	95	2	0.39	0.39	0	1
10.1	F	L	NS	Neg	6	11	15	70	2	0.12	0.50	43.75	65	2	0.60	0.46	8	2
10.2	-	R	-	-		13	18.25	97	2	0.82	4.02	16.25	98	1	1.41	5.04	−25	7
11.1	M	L	FS	Neg	12	12	20	100	1	NA	0.67	10	100	2	0.08	0.26	61	3d
11.2	-	R	-	-		15	10.5	100	2	0.87	3.15	130	0	5	0.06	0.27	92	4
12.1	M	L	FS	Neg	1	10	5	100	1	0.16	0.36	10	100	2	0.52	0.25	29	3b
12.2	-	R	-	-		13	11.25	98	2	0.05	0.30	12.7	96	2	0.56	0.30	2	2
13.1	F	L	Large genome alteration	Pos	3	10	5	100	1	0.02	0.11	10	100	1	0.14	0.07	39	3c
13.2		R	-	-		11	11.25	98	2	0.21	0.56	12.7	96	2	0.37	0.44	22	3b

14.1	F	L	NO	Neg	22	22	5.5	95	2	0.93	1.26	23	81	1	0.07	0.08	94	4
14.2	-	R	-	-		23	43	69	1	0.33	0.58	45	5	4	0.13	0.07	88	3e
15.2	F	R	FS	Neg	13	18	10.75	95	3	0.45	5.96	47.5	95	3	2.93	4.06	32	3c
16.1	F	L	SP	Neg	22	24	12.5	99	2	1.08	3.65	25.25	100	2	−0.15	1.67	54	3d
16.2	-	R	-	-		23	15	100	2	1.07	4.03	24.6	80	2	−0.22	2.48	39	3c
17.1	M	L	MOS	Neg	26	30	25	100	3	1.52	5.75	22	70	3	0.69	4.91	15	3b
17.2	-	R	-	-		30	11.3	90	3	0.79	3.57	25	70	5	0.36	0.68	81	3e
18.2	M	R	NS	Neg	6	15	28.75	100	1	0.08	0.52	130	0	5	0.07	0.20	61	3d
19.2	F	R	DEL	Neg	7	12	6.25	98	1	0.07	0.25	4	100	1	0.29	0.17	33	3c
20.1	F	L	DEL	Neg	16	17	53.75	1	2	NA	10.53	130	0	5	−0.02	0.26	98	5
20.2	-	R	-	-		25	80	5	2	1.48	19.50	130	0	5	0.50	8.76	55	3d
21.2	M	R	NA	Neg	22	18	15	100	2	0.61	2.00	20	100	3	0.61	0.76	62	3d
22.1	M	L	SP	Neg	10	13	12.5	100	2	0.05	0.21	10	90	2	0.27	0.12	42	3c
22.2	-	R	-	-		12	6.25	95	2	0.33	1.00	6.25	100	2	0.24	0.50	50	3d
23.1	F	L	FS	Neg	14	15	6.5	90	2	0.60	1.71	7.5	100	2	0.30	0.86	50	3d
23.2	M	R	-	-		16	5	100	2	0.20	0.50	17	100	2	0.02	0.25	50	3d
24.1	M	L	SP	Neg	13	14	16.25	99	1	0.63	1.25	23.75	100	2	0.06	1.04	16	3b
24.2	-	R	-	-		14	14.5	100	2	0.03	0.66	6.25	100	1	0.24	0.56	15	3b
25.1	M	L	SP	Neg	15	16	5	100	2	0.15	0.61	1.28	100	2	0.04	0.72	-18	7
25.2	-	R	-	-		16	1.25	100	2	2.72	5.89	3.25	100	2	0.08	0.80	86	3e
26.2	F	R	SP	Neg	21	26	8.75	100	2	0.34	1.63	8	100	2	0.31	0.92	44	3c
27.2	F	R	SP	Neg	36	36	8.75	100	3	NA	3.39	130	0	5	0.03	0.55	84	3e
28.1	F	L	MOS	Pos	22	23	48.75	58	2	1.71	8.98	47.3	5	2	NA	8.22	8	2
28.2		R	-	-		24	12.5	100	2	0.23	8.96	18.75	90	2	4.25	8.55	5	2
29.1	F	L	MOS	Neg	29	34	55	70	1	0.01	0.26	58.5	70	1	NA	0.25	5	2
30.2	M	R	NA	Neg	32	40	25	85	1	0.03	0.43	31	80	1	0.09	0.06	86	3e
31.1	M	L	FS	Neg	18	18	31.25	83	2	NA	11.05	130	0	5	−0.01	0.16	99	5
31.2	-	R	-	-		22	8.75	95	2	0.54	2.92	130	0	5	0.54	2.47	15	3b
32.2	M	R	NA	Neg	16	25	32.5	80	2	NA	2.54	25	100	4	0.68	2.42	5	2
33.2	F	R	NA	Neg	22	23	35	30	4	1.82	1.89	30	85	4	0.01	0.24	87	3e
34.1	M	L	SP	Neg	7	20	16	96	3	0.23	1.66	18.75	99	3	1.42	1.44	13	3b

34.2	-	R	-	-		20	11.25	100	3	0.45	3.22	19	99	2	−0.01	1.95	39	3c
35.2	M	R	NA	Neg	14	16	11.25	100	1	NA	0.61	22	98	1	NA	0.51	16	3b
36.1	F	L	SP	Neg	11	15	5	100	2	0.21	0.85	5	95	2	NA	1.06	−25	7
37.1	M	L	MOS	Neg	13	30	8.75	100	1	0.09	0.55	8.25	100	1	0.04	0.07	87	3e
38.1	M	L	MOS	Neg	19	32	1.25	99	2	0.85	7.21	2.67	100	2	−0.03	0.56	92	4
39.2	M	R	NA	Neg	NA	22	55	40	2	NA	20.51	55	47	2	4.54	2.49	88	3e
40.1	F	L	SP	Neg	16	16	11.25	90	2	10.84	33.92	6.25	65	4	19.84	14.68	57	3d
41.1	F	L	NA	Neg	24	29	22.25	100	2	NA	2.64	25.25	99	2	NA	1.32	50	3d
42.1	M	L	NA	Neg	32	32	62.5	100	2	0.04	0.65	68.5	80	2	0.50	0.49	25	3b
42.2	-	R	-	-		33	64.25	90	2	0.08	1.66	70.5	35	5	−0.08	0.54	67	3d
43.1	M	L	SP	Pos	8	25	39.5	60	3	0.04	1.53	28	59	3	NA	1.49	3	2
43.2	-	R	-	-		12	2.5	100	2	NA	NA	1.75	100	2	0.34	1.17	NA	NA
44.1	M	L	NS	Neg	20	39	43.75	100	2	0.16	2.32	45	100	3	0.28	2.89	−25	7
45.1	F	L	NA	Neg	18	33	40	70	4	NA	NA	45	60	4	0.58	2.23	NA	NA
46.1	F	L	NA	Pos	25	47	52	44	5	0.32	28.12	60	40	5	0.04	2.98	89	3e
47.2	M	R	NA	Neg	NA	22	10	100	2	NA	25.30	130	0	5	−0.03	0.17	99	5
48.1	F	L	NA	Neg	NA	27	31	80	2	NA	15.52	56	70	2	NA	6.23	60	3d
49.1	F	L	NA	Neg	18	19	26.25	85	1	NA	3.08	130	0	5	0.03	0.01	100	6
49.2	-	R	-	-		22	10	100	2	0.36	1.31	11.25	100	2	0.11	0.66	50	3d
50.2	M	R	MOS	Neg	29	40	18.75	98	1	1.29	5.24	24.5	94	2	0.36	0.97	81	3e
51.1	M	L	NA	Neg	16	18	11.25	100	2	NA	3.32	20	90	3	0.19	1.98	40	3c
52.2	M	R	NA	Neg	22	28	33.75	95	2	NA	3.20	44	90	3	0.25	0.96	70	3e
53.1	M	L	MIS	Pos	37	46	45	60	2	-0.08	4.24	47.5	65	3	0.04	2.19	48	3c
53.2	-	R	-	-		46	21.25	90	2	NA	0.89	35	70	3	−0.01	0.81	9	3a
54.1	M	L	DEL	Neg	15	27	50	100	3	0.36	5.72	47.5	85	4	0.34	2.13	63	3d
55.2	M	R	FS	Pos	6	21	10	98	1	0.00	0.25	6.25	100	1	NA	0.13	48	3c
56.1	F	L	FS	Pos	14	26	25	90	2	NA	6.78	55.25	60	2	0.07	0.37	95	5
56.2	-	R	-	-		21	49.5	50	3	0.00	NA	64.75	40	2	5.77	2.35	NA	NA
57.1	F	L	FS	Pos	NA	20	12.5	100	2	NA	1.49	15	99	2	NA	NA	NA	NA
58.1	F	L	FS	Neg	13	14	33	95	2	0.51	2.27	32	90	2	0.11	1.87	18	3b
58.2	-	R	-	-		14	37	100	2	2.95	8.13	38	100	2	1.05	4.69	42	3c

59.2	M	R	NA	Pos	19	23	21.75	100	1	6.74	24.50	130	0	5	1.12	1.18	95	5
60.1	M	L	NO	Neg	NA	30	8.75	100	1	0.01	0.14	9	100	1	0.25	0.00	100	6
60.1	M	L	-	-		25	35	80	2	0.21	1.92	40	70	2	NA	0.00	100	6
61.1	F	L	NA	Neg	4	22	12	92	2	0.02	0.59	16	90	1	0.02	0.58	2	2
62.1	M	L	NA	Neg	27	31	59.75	50	4	0.82	9.23	130	0	5	0.05	1.21	87	3e
63.1	F	L	NA	Neg	13	16	15	90	2	1.44	4.45	20	90	2	0.86	3.90	12	3b
63.2	-	R	-	-		16	26	1	2	2.60	8.18	35	1	4	2.60	3.03	63	3d
64.2	F	R	MOS	Pos	41	42	9	100	2	0.42	0.60	130	0	5	0.00	0.00	100	6
65.1	M	L	NA	Pos	27	27	81.25	60	5	14.96	33.14	130	0	5	0.00	0.08	100	5
66.2	M	R	NA	Neg	31	40	28	80	1	0.03	0.34	33.75	70	1	0.03	0.06	83	3e
67.1	F	L	NS	Neg	14	22	16.25	100	1	0.73	21.94	18.75	100	3	0.83	18.05	18	3b
68.1	M	L	FS	Pos	11	14	10	100	1	0.16	0.68	13.75	100	2	0.72	0.78	−15	7
68.2	-	R	--			15	20.75	90	2	0.10	0.58	25.75	90	2	0.00	0.70	−22	7
69.2	M	R	FS	Pos	16	29	17.5	90	2	0.00	0.30	33.75	85	2	NA	0.21	31	3c
70.2	F	R	NO	Neg	26	27	17	80	3	0.44	0.84	33.75	80	2	−0.21	0.74	12	3b
71.2	M	R	NA	Pos	21	24	30	80	1	0.01	0.20	27.5	85	1	NA	0.03	87	3e
72.2	M	R	FS	Neg	11	19	36	60	1	0.02	0.33	36.25	75	2	NA	NA	NA	NA

PTA—pure-tone average; SDS—speech discrimination score; BAEP—brainstem auditory evoked potentials Classification System according to Samii and Matthies et al.[1]; F—female; M—male; L-left; R—right; NA—not available; NA—not available; F—female; M—male; NS—nonsense mutation ; FS—frameshifting mutation; MOS—mosaic, MIS—missense mutation; DEL—deletion; NO—no mutation found neither in blood nor in tumor DNA. Pos—positive family history of NF2; Neg—negative family history of NF2. *Resection amount categories are described in the main text (Table 2). Resection amount in % was calculated with pre- and postoperative volume values. In all patients, a decompression of the internal auditory canal (IAC) was performed. Preoperative values: measured directly or within 4 weeks before surgery. Postoperative values: measured directly or up to 3 months after surgery. Tumors/cases with large discrepancy between preoperative BAEP and audiometry (AAO-HNS class) ≥ 2 points are highlighted in gray, bold and italics.

References Supplementary Materials

1. Matthies, C.; Samii, M. Management of Vestibular Schwannomas (Acoustic Neuromas): The Value of Neurophysiology for Evaluation and Prediction of Auditory Function in 420 Cases. *Neurosurg.* **1997**, *40*, 919–930, doi:10.1097/00006123-199705000-00007
2. Committee on Hearing and Equilibrium Guidelines for the Evaluation of Hearing Preservation in Acoustic Neuroma (Vestibular Schwannoma): Committee on Hearing and Equilibrium. *Otolaryngol. Neck Surg.* **1995**, *113*, 179–180, doi:10.1016/s0194-5998(95)70101-x.
